# Patients’, Carers’, and the Public’s Perspectives on Electroconvulsive Therapy

**DOI:** 10.3389/fpsyt.2019.00304

**Published:** 2019-05-07

**Authors:** Chris Griffiths, Alex O’Neill-Kerr

**Affiliations:** Innovation and Research Department, Northamptonshire Healthcare NHS Foundation Trust, Northampton, United Kingdom

**Keywords:** depression, electroconvulsive therapy, electroconvulsive, therapy, perspectives

## Abstract

**Aims and Method:** The aim of this study was to present patients’, carers’, and the public’s perspectives on electroconvulsive therapy (ECT) through a narrative review of the literature.

**Results:** People’s perspectives on ECT are often negative due to media and Internet portrayal. Perspectives are influenced by risks, short-term side effects, and the most commonly reported longer-term side effect: memory loss. However, many patients do not report memory loss. Most people who experience ECT and their carers report a positive perspective. In the future, people’s perspectives may become more positive with higher service delivery standards and a more balanced, well-informed view of modern ECT presented by the media. However, ECT has risks and side effects, and negative and critical perspectives on the use and effects of ECT will persist.

**Clinical Implications:** Perspectives on ECT are important because of the impact on stigma, patient treatment choice, patient consent, and provision of and referral for ECT.

## Introduction

Information about electroconvulsive therapy (ECT) that is not representative of current practice, evidence, and experience can distort people’s perspectives. The public’s perspectives on ECT are important because they can be influential on attitudes to patients who receive treatment and stigma and discrimination associated with receiving ECT. Perspectives of people who might benefit from receiving ECT and existing ECT patients are important because they can determine choice of treatment, consent to treatment, and self-stigma. Perspectives of carers can influence patient choice of treatment and consent to treatment and the support that they provide a patient. Together, these perspectives can affect the demand for and availability of ECT. This paper reviews published academic journal and nonacademic journal-based literature on patients’, carers’, and the public’s perspectives on ECT.

## Methods

A narrative review was undertaken. The online database Google Scholar was used to search for eligible studies. Google search engine was used to search for nonacademic journal-based literature. Search terms included the following: “ECT,” “electroconvulsive therapy,” “perspectives,” “subjective,” “view,” “attitude,” “opinion,” and “experience.” Studies and articles found were collated and reviewed to extract content related to the topic of the narrative review.

## Results

### Public’s Perspectives

The public’s perspectives of ECT depend on many factors: what they read, see, and hear through the media and social media or the knowledge gained through friends and family if they have had experience of ECT. Public perceptions of ECT are mainly negative (1). The portrayal of ECT in books and films, for example, *One Flew Over the Cuckoo’s Nest* (1975), *Frances* (1982), *Any Angel at My Table* (1990), and *Shine* (1996), is almost exclusively negative. A 2001 review of all films featuring ECT reported that films have become progressively more negative about ECT, portraying it as cruel, brutal, harmful, and abusive, with little therapeutic benefit (2). This portrayal is influential on the public’s opinions of ECT (3, 4). It can also create fear of ECT in doctors (5). Portrayal in films rarely depicts modern ECT practice and experience; therefore, it distorts public perspectives.

The Internet is a source of both impartial and false or distorted information related to ECT, meaning that it is not easy for people to discern the truth (6). Inaccurate information biased against ECT generates negative opinions and belief systems, which can lead to societal stigma toward individuals undergoing or having undergone ECT, possibly leading to discrimination (6). The impact of this negative portrayal in the media and on the Internet can also lead to self or internalized stigma in people who have had ECT.

There is a lack of media coverage countering the negative portrayal of ECT (6); however, more recently, we have seen descriptions of personal accounts of ECT available on the Internet, for example, on the website Technology Entertainment Design and in autobiographies such as “Electroboy” (7, 8). Newspapers have described modern ECT practices and also published more recent personal experiences of ECT, describing positive aspects both from carer and patient perspectives (9–11). It is important to counter ill-placed public perception that ECT is anarchic and barbaric, severely damages those who undergo it, and provides little therapeutic benefit (6). The media, healthcare professionals, people who experience ECT, and those who care for them have a role to play in ensuring that accurate descriptions and portrayals of modern ECT are represented in the media and on the Internet, that balanced views both of positive and negative aspects of ECT are portrayed.

### Patient Perspectives

Patient and carer perceptions of ECT are also influenced by media portrayal. Patient’s perspectives are not passively generated; patients are actively making sense of their experience before, during, and after treatment (12). Patient’s perspective of ECT is more complex than simply its efficacy in reducing the symptoms of depression; perspectives encompass fears, stress before and during treatment, possible side effects (especially memory loss, confusion, loss of cognitive ability), stigma, and regaining a sense of self and reality (13). Patient perspectives have been the subject of several research studies.

Surveys of patients who have undergone ECT treatment have revealed positive attitudes to its effectiveness (14–16). In terms of the experience of ECT, in one study, less than a fifth of respondents rated ECT as slightly as or much worse than going to the dentist, and most (97%) did not report the experience to be very stressful (14). Support for further sessions of ECT have been reported as being high (16). However, the majority of patients hold the view that relief of depressive symptoms is short lived and that repeated treatments are required (17).

Perspectives can be influenced by the information given before and after treatment. A review of patient experience revealed high rates of unsatisfactory pretreatment information, feelings of treatment coercion, and unallayed fears (18). Negative perspectives that exist toward ECT might be partially attributed to inconsistent standards surrounding patient information and consent processes (18, 19). This highlights the importance of effective patient engagement with knowledge about ECT and empowering patient involvement in treatment choice (18).

There have been improvements over time in pretreatment information, allaying fears, patient engagement, reducing feelings of coercion, and involving patients in treatment choice. A 1976 review revealed that only 21% of patients reported that they were given adequate information prior to treatment (20). Subsequently, a 2004 study found that around 80% of patients stated that the treatment had been fairly or very well explained (14), with 85% in a 2007 study stating that written information was helpful (15). Fears about ECT treatment can be alleviated and perspectives changed if a patient has the process and treatment fully explained to them by medical staff (21).

The ECT accreditation service of the Royal College of Psychiatrists (ECTAS) and the Scottish ECT accreditation network have undertaken a series of initiatives to improve the quality of information that is given to patients prior to ECT. The ECTAS 2013 to 2015 report noted that “there have been significant improvements in practice since the inception of the scheme” (22). There are increased numbers of clinics accredited as excellent, with several clinics meeting 100% of standards. The annual report also detailed the improved input of patient’s views and refers to a number of initiatives that aim to make the patient experience more central to the accreditation process.

A total of 202 patient questionnaires were returned to ECTAS and feedback was generally positive, for example, 85.5% of people answered “yes” to the question “did your doctor speak to you about ECT before you agreed to treatment?”, only 3.5% of patients said “no,” the remainder responded “don’t know/can’t remember,” and 88% of people agreed that clinic staff were friendly and reassuring. Free-text comments often praised the quality of care and the friendliness and caring attitude of staff. In terms of the effectiveness of ECT, the ECTAS annual report noted that 77% of people responded “yes” to the question “did ECT help you?”; only 12% of people said “no” and the remainder responded “don’t know/can’t remember.”

A meta-analysis of eight longitudinal studies showed that experiencing ECT both increased patient’s knowledge of and improved attitudes toward ECT (23). In the majority of cases, patients who have undergone ECT report positive attitudes toward the treatment (18, 20, 24–26). An analysis of routinely collected anonymous ECT NHS patient feedback highlighted the importance of positive interactions with staff on perspectives, satisfaction, and experience (27). This NHS Trust feedback is made public, therefore providing information on patient’s experience of ECT to patients, caregivers, prescribers, and anyone else who chooses to read it; this is a model that can be replicated throughout the NHS.

A key aspect of patient perspectives about ECT is their experience of any side effects of undergoing the treatment. Although neurological tests used in ECT studies have shown little evidence of persistent memory loss, these tests tend to measure ability to form new memories, whereas patients report erasing of autobiographical memories or retrograde amnesia (19). Memory loss is commonly reported as a side effect that can significantly impact on patient’s lives; however, it is a complex issue due to the association between depression and memory impairment (28).

Various studies have reported subjective rates of memory loss: 30 patients were interviewed and 80% reported memory loss, 389 respondents were surveyed and 50% reported memory loss, 51 patients were surveyed and 60% reported memory impairment, and 108 patients were questioned and 45% reported persistent memory loss (15, 16, 29, 30). However, a review of subjective memory complaints—assessed mostly on the Squire Subjective Memory Assessment—found the majority of studies reporting improved subjective memory after ECT (31). Some research indicates that the more courses of ECT someone undergoes, the more it is likely to affect their memory; however, this has been disputed (32, 33). There have been recent recommendations that assessment of cognitive function following ECT is conducted in all routine clinical practice (31, 34).

There are also short-term side effects that may occur immediately after treatment, including drowsiness, confusion, headache, sickness, and aching muscles (29, 30). Both immediate and longer-term side effects can negatively impact on patient’s perspectives of ECT, but patients are likely to consider that they are acceptable if they are outweighed by the antidepressive benefits of ECT; patients engage in a “cost–benefit analysis” (16). Despite reported side effects, the majority of patients find ECT to be beneficial, would recommend ECT to others, and would have it again (29, 35). Sixty-eight patients who had undergone ECT treatment were interviewed and the participants viewed ECT as an effective and legitimate treatment (36).

### Carer Perceptions

Carer perspectives are important because they can play a role in the consent process and can be influential in patient choice of treatment (12). There has been very little research conducted in terms of carer perspectives of ECT. In one study where seven family members were interviewed about being involved in the process of making decisions about treatment, participants reported feelings that “ECT was the last hope” and that “blind trust that had to place in the doctor” (17). Twenty-eight parents of young people (aged less than 19 years who had received ECT) were interviewed and 17 thought that the treatment was helpful, 9 thought it made no difference, and 1 thought it was harmful (35). In another small study, family members had highly positive attitudes to ECT both before and after treatment and, following treatment, felt strongly that ECT was beneficial (37).

Twenty-seven carers of people who had undergone ECT were interviewed about their experience and attitudes to ECT (29). The majority expressed the view that ECT was beneficial and would support their family member again to undergo ECT. Some carers’ perspectives were that ECT should only be used as a treatment of last resort due to associated memory problems (29). In an analysis of five longitudinal studies of family members, it was found that, following ECT treatment, there was a significant positive change in carer knowledge of ECT but no significant change in attitudes toward ECT (23).

In 2015, ECTAS introduced a self-review focusing on the experiences of relatives, friends, and carers (22). Every clinic that begun their self-review in 2015 has been asked to distribute copies of this questionnaire, and these are returned directly to ECTAS. As of October 2015, 36 carer questionnaires had been returned by 14 clinics. Early results suggest that carers are generally pleased with the care that their relative or friend has received at the clinic. The following is illustrative: “I was very pleased with the standard of care given to my relative at the ECT clinic. They treated her with dignity and respect and were very kind to us both.”

## Conclusion

ECT is an antidepressant treatment used when other less intrusive treatments have failed. ECT is still the most effective treatment for severe treatment-resistant depression (38). It is a treatment that is delivered in a hospital environment, causes a seizure, involves anesthesia, requires the use of a bite block, often requires repeated treatment due to short lived effect, and has side effects (some of which may be long-term for a minority of patients). Historic negative portrayal in the media does not align with a more positive patient and carer perspective of ECT delivered by NHS services today. Public, patient, and carer perspectives may improve with higher standards of service delivery and a more balanced and well-informed view of modern ECT presented by the media and other sources; however, critical and negative perspectives will remain.

## Author Contributions

CG wrote the article. AO reviewed the article, contributed text, and added references.

## Funding

The authors received support through the Northamptonshire Healthcare NHS Foundation Trust.

## Conflict of Interest Statement

The authors declare that the research was conducted in the absence of any commercial or financial relationships that could be construed as a potential conflict of interest.
